# The American Journal of Insanity

**Published:** 1847-10-01

**Authors:** 


I. The American Journal of Insanity. April, 1847.

II. Fourth Annual Report of the Managers of the State Lunatic Asylum, New York, 1847.

III. Remarques sur quelques Etablissemens d'Aliénés de la Belgique, de la Hollande, et de L'Angleterre. Par M. *Brierre de Boismont*. [Annales d'Hygiène Publique, T. 37 & 38. 1847.]

Remarks upon some of the Establishments for the Insane in Belgium, Holland, and England. By M. *Brierre de Boismont*.

Having so recently dwelt upon the various topics connected with the Insane, we should not have again adverted to the subject at present, but that the above-named publications, which are not likely to fall into the hands of many of our readers, contain some well-written papers, of which an account can scarcely fail to prove interesting. In the Journal of Insanity, Dr. Ray, Superintendent of the Butler Hospital for the Insane, whose Essay upon the British Lunatic Asylums we recently noticed, has an excellent critical dissertation upon—

*Shakespeare's Delineations of Insanity.*

After observing that none but a master-mind can, from a few casual opportunities, present a portrait of the pathological conditions of the human mind with sufficient faithfulness to satisfy the professional observer, he goes on to say:—

" Such a mind was Shakespeare's; and it is because he clearly perceived at a glance those numberless shades of distinction that entirely escape the notice of ordinary observers; that his characters, whether sane or insane, are neither personified abstractions of specific qualities, marked by a name and assigned a part in the play; nor servile copies from life that have lost their interest under the process of transference, but real, mortal men, who live and act before us, and lose their senses it may be, and whose names live after them in the memory of men. His success in this difficult line is to be attributed to that distinguishing faculty of his mind of deducing with wonderful correctness general principles of character from the narrowest possible range of observation. And yet he had peculiar difficulties to overcome. He had not only to divest himself of the popular misconceptions of insanity which regard it as a jumble of intellectual manifestations, acknowledging no principle of cohesion or concatenation, but his opportunities for observing the insane were scanty and imperfect. No friendly asylum furnished subjects for study, whose mental endowments were worthy of his study, and such as he occasionally met at the road-side, or beheld through the bars of their prison-house, were for the most part, it is probable, too far degraded by neglect and unkindness, to be conducive to any poetical purpose. It is not to be supposed, however, that he was guided solely by intuition. He unquestionably did observe the insane, but he observed them as the great comparative anatomist of our age observed the remains of extinct species of animals —from one of the smallest bones, re-constructing the whole skeleton of the creature, re-investing it with flesh and blood, and deducing its manners and habits. By a similar kind of sagacity, Shakespeare, from a single trait of mental disease that he did observe, was enabled to infer the existence of many others that he did not observe, and from this profound insight into the law of psycological relations, he derived the light that observation had failed to supply. Thus, in spite of all the obstacles in his way, he succeeded to a degree that has seldom been equalled, in representing insanity, both in the form of maniacal wildness and disorder, and that of melancholy dejection and gloom. Its progress through various stages, from the first scarcely perceptible deviation from the soundness of health to its termination in recovery or death, is traced with that thorough fidelity to nature so characteristic of all his conceptions." P. 291.

And thus it is : the more diligently that Shakespeare is studied by the aid of the lights which modern investigations and improvements have shed upon the normal and morbid conditions of the human mind, the more must we arise from such study impressed with admiration at the surprising insight into its innermost workings which his genius supplied him with. Much trash has indeed been set forth under the guise of commentary upon his immortal dramas ; and passages in his writings have been twisted and turned to indicate the anticipation of numerous discoveries appertaining solely to a later epoch ; but the more they are examined by the aid of reason, knowledge, and experience, rather than of the imagination, the more impressed shall we become with the persuasion that an intuitive knowledge of the laws of thought, and the springs of action of the human mind, was his portion in a degree possessed by no man prior or subsequent to his epoch. Perhaps in no case could this be better exemplified than in his mode of delineating insanity—the mere vulgar idea of which is so untrue and so easily realized, while the nicer touches escape all but the master's eye and hand.

Dr. Ray commences his Essay with an account of the madness of Lear, the picture of which he regards as a master-piece. Lear is eminently predisposed to insanity, being a man of "hot and hasty temper, though endowed with strong and generous passions, of a credulous and confiding disposition, governed by impulses rather than deliberate judgment, rendered impatient of restraint or contradiction by the habit of command, with a nervous temperament, strongly susceptible of the vexations of life, and moreover, with all those moral infirmities aggravated by old age." The early stage or incubation of the insanity is manifested not so much by marked delusions, as by an exaggeration of these peculiarities of character: and we are quite prepared to expect that the mind, which was so ill counterpoised as to banish his favourite child and abdicate his throne from mere unreflecting impulse, will sink under the infliction of the ingratitude of his other daughters, upon whom he had conferred so much: but the effects of this, as displayed in the generous confiding old man's credulity at first, and then his intolerable anguish of mind, mingled with the conviction that his senses are forsaking him, form a series of pictures, which for beauty and truth to nature are unrivalled.

"Unable as the insane are to perceive their own insanity, yet this apprehension of its approach, so frequently repeated by Lear, usually occurs during its incubation. While still able to control his mental manifestations, the patient is tortured with anticipations of insanity, but when he actually becomes so insane, that the most careless observer perceives the fact, then he entertains the most complacent opinion of his intellectual vigour and soundness. And yet this is one of the nicer traits of insanity which the ordinary observer would hardly be supposed to notice." P. 297.

Dr. Ray observes that Shakespeare has exhibited additional proofs of his observant powers in his delineation of the raving stage of Lear's insanity. This in the ordinary acceptation indicates but a mere arbitrary unconnected jumble of words; but, in point of fact, the ideas which these represent are connected by certain laws of association, although the discovery of them may be no easy matter. During the cerebral excitement, impressions formerly made may be vividly recalled, so as to resemble present realities, and as such are thought and discoursed about by the patient. Such images, too, rapidly succeed each other, and give rise to corresponding ever-changing expression of the thoughts in mania. The condition is very analogous indeed to that of ordinary dreaming, and "mania may with some propriety be designated as dreaming with all the senses open, the morbid excitement rendering the images unusually vivid." Again, the maniac, unrestrained by any sense of propriety or fitness, gives expression to any thought that may present itself; while, in a person of sane mind, a large portion of the thoughts remain unuttered on account of their want of relevancy to the subject in hand. "Every one must be aware how often, in the course of ordinary conversation, thoughts start up having the remotest possible connexion with anything already said—so remote indeed as to defy any one but himself to discover it. Any person who should utter every thought that arose in his mind, in the freest possible conversation, would most certainly be taken for a fool or a maniac." Bearing these facts in mind—

"We readily see how there should always be some method in madness, however wild or furious it may be; some traces of that delicate thread, which, though broken in numerous points, still forms the connecting link between many groups and patches of thought. It is in consequence of Shakespeare's knowledge of this psycological law, that in all his representations of madness, even though characterized by wildness and irregularity, we are never at a loss to perceive that the disease is real, not assumed. Not so, however, with most writers, even of distinguished name, who have undertaken to represent the workings of a raving mind. Unaware of the law in question, and governed by the popular notions upon the subject—they seem only to have aimed at unlimited extravagance and incoherence." P. 302.

Lear's recovery from his insanity is depicted with no less consummate skill. He awakes recovered from a refreshing sleep, which the drugs administered to him have procured, but it is at first but dimly and only gradually he comprehends his real position.

"A more faithful picture of the mind when it is emerging from the darkness of disease into the clear atmosphere of health restored, was never executed than this of Lear's recovery. Generally, recovery from acute mania is gradual, one delusion after another giving way, until after a series of struggles which may occupy weeks or months, between the convictions of reason and the suggestions of disease, the patient comes out a sound, rational man. In a small proportion of cases, however, this change takes place very rapidly; within the space of a few hours or a day, he recognizes his true condition, abandons his delusions, and contemplates all his relations in an entirely different light." P. 304.

As Edgar, in the same play, simulates insanity, it furnishes the poet with an opportunity which he does not neglect to avail himself of, of exhibiting the exaggeration which would be likely to take with the multitude, but not to deceive the experienced observer. "Had it been Shakespeare's design to exhibit a case of real demonomania, or of chronic mania, we should unquestionably have had something very different from the part of Edgar. If the former, we should not have found the patient talking so clearly about his own case, while indulging in unlimited incoherence and rambling about every thing else; and if the latter, we should not have seen a strain of acute morality succeeded, more than once, by a trait of mental imbecility."

Dr. Ray devotes a large space to the consideration of that noblest of all plays, HAMLET, and brings forward cogent reasons for gainsaying the gratuitous conclusions of those commentators who maintain the prince's insanity was feigned. Into his analysis of these opinions, founded as they are for the most part on an imperfect apprehension of the nature of the disease, we have not space to enter: but certainly the difficulties which are supposed to attach to the interpretation of some portions of the drama seem to us most easily soluble upon the hypothesis of the reality of the insanity. The true character of Hamlet's madness is thus set forth:

"In Lear we are presented with the origin, progress, and termination of a case of acute mania—that form of mental disorder in which the mind becomes, at last, completely unsettled, and all its operations pervaded by discord and confusion. Hamlet's insanity differs from Lear's, in not having the successive steps of its progress so well-marked and regular; in presenting less incoherence of thought, and less nervous excitement. In his case, acute general mania like Lear's would have been incompatible with that degree of forecast and self-control which the character required; and simple monomania, where the sphere of the aberration is a very limited one, the individual, for the most part, observing the ordinary proprieties and courtesies of life, would have been equally out of the question, because it would not have exerted the requisite influence over the action of the play. With great skill, therefore—a skill founded on what would seem to be a professional knowledge of the subject—Shakespeare has selected for his purpose that form of the disease in which the patient is mad enough to satisfy the most superficial observer, while he still retains sufficient power of reflection and self-control to form and pursue, if not to execute, a well-defined, well-settled purpose of revenge. In order the better to understand the conduct of Hamlet, we should bear in mind that he was a man of warm affections, refined tastes, and a quick sense of honour, and possessed of a high order of intellectual endowments. With these simple elements of character the manifestations of disease are made to harmonize and blend so intimately together, that it is not always easy to distinguish between them." P. 313.

Rendered sad and gloomy by the death of his father and the marriage of his mother, the subject of suicide had already anxiously occupied his thoughts, when the bloody secret revealed to him by his father's ghost induces a temporary flightiness and levity of manner too plainly indicative of its disordering effect upon his faculties. His subsequent interviews with Ophelia and Polonius are markedly indicative of insanity; while before his friends Rosencrantz and Guildenstein, who he suspected were bent upon watching him, he makes no attempt to prove the existence or reality of his madness—favourable as the opportunity was for simulation. He calmly describes to them the perverted condition of his feelings which had of late distressed him.

" 'I have of late (but wherefore I know not) lost all my mirth, foregone all custom of exercises, and, indeed, it goes so heavily with my disposition, that this goodly frame, the earth, seems to me a sterile promontory; this most excellent canopy, the air, look you, this brave o'er-hanging firmanent, this majestical roof fretted with golden fire, why, it appears no other thing to me than a foul and pestilent congregation of vapours.' A most faithful and vivid picture is this of a mental condition that is the precursor of decided insanity—the deepening shadow of that steadily advancing eclipse by which the understanding is to be darkened. In Hamlet the disease has not yet proceeded so far as to prevent him, in his calmer moments, from recognizing and deploring its existence, though he mistakes its character. Like every other person in his condition, he is very far from considering himself insane, and indeed there is no reason why he should. He entertains no delusions; persons and things appear to him in their customary relations; and, for the most part, he well sustains his character as a man and a prince. His unwonted excitability of temper, his occasional disregard of some minor propriety of life, the cloud which envelopes all outward things, depriving them of their worth and beauty—in the eyes of the world these do not constitute insanity, and are not incompatible with the most perfect integrity of intellect. Why, then, should he suppose himself insane or beginning to be so? Such a mistake is very natural to the patient, but when made by others who vaunt their knowledge of mental pathology, it proceeds from a less excusable kind of ignorance." P. 318.

The test of his sanity which Hamlet proffers in answer to the charge of madness—

" It is not madness
That I have uttered; bring me to the test,
And I the matter will re-word which madness
Would gambol from,"

is undoubtedly satisfactory enough in many cases, and was employed and commented upon with ability by Sir Henry Halford; but as Dr. Ray truly observes, that although "in most cases of *acute mania*, attended with much excitement, as well as in that form of mental impairment called *dementia*, the patient would be unable, no doubt, to repeat what he had just before deliberately uttered, but in such cases as Hamlet's, where some of the mental operations are perfectly well conducted, the power of repeating correctly one's own statements is not necessarily lost, and consequently is no proof of sanity in doubtful cases."

In allusion to that beautiful creation in the same play, the insanity of Ophelia, in consequence of the utter wreck of her hopes and affections, Dr. Ray observes—

" In this play, for the first and only time, Shakespeare has ventured on representing the two principal characters as insane. His wonderful success in managing such intractable materials the world has long acknowledged and admired. They are never in the way, and their insanity is never brought forward in order to enliven the interest by a display of that kind of energy and extravagance that flows from morbid mental excitement. On the contrary, it assists in the development of events, and bears its part in the great movement in which the actors are hurried along as if by an inevitable decree of fate. Herein lies the distinguishing character of Shakespeare's delineations of insanity. While other poets have made use of it chiefly to diversify the action of the play, and to excite vulgar curiosity by its strange and striking phenomena, he has made it the occasion of unfolding many a deep truth in mental science, of displaying those motley combinations of thought that are the offspring of disease, and of tracing those mysterious associations by which the ideas of the insane mind are connected. Few men, I apprehend, are so familiar with those diversities of mental character, that are in any degree the result of disease, as not to find the sphere of their ideas on this subject somewhat enlarged by the careful study of Shakespeare." P. 324.

We have not space to accompany our author in his examination of the hallucinations of Macbeth and the shattered mental constitution of his ambitious wife. After alluding again to the inferiority and inartistic characters of the delineations of insanity by other dramatists, in which all individuality of character is merged in the mere vulgar and conventional idea of insanity, with all its exaggerations and errors, he thus concludes his interesting Essay.

"In this review of Shakespeare's Delineations of Insanity, I trust I have made it appear in some measure how their wonderful fidelity to nature renders them not only valuable as pathological illustrations, but wonderfully effective in producing a dramatic impression. Great as he is in every other attribute of the poetical character, yet in this department of the art, he seems to be without a rival. No other writer, unless we except Sir W. Scott, has made the slightest approach to his success. In several of this writer's works, the working of a disordered mind are displayed with the hand of a master, and that too with a degree of pathological accuracy which ordinary men would hardly acquire by years of observation within the precincts of a hospital. But the moralist possesses an advantage over the poet in the broader limits within which he may exercise his art, untrammelled by the restrictions enforced upon the other by severer rules of composition and the comparative brevity of his efforts.

"I have already intimated that, in his knowledge of insanity, Shakespeare was greatly in advance of his own and succeeding generations; and that this was owing not to any superior advantages he possessed for the study of the disease, but to an extraordinary power of observation which more than any other mental attribute, perhaps, deserves to be considered as the true inspiration of genius. It needs but a glance at the common views of insanity that prevailed in his own, and even later times, not merely among the rude and uneducated, but among men of distinguished names, to show how little they evince of his profound science of mind. By a profession which has always numbered in its ranks a large proportion of the luminaries of the age, the insane generally, with the exception of such as were actually raving or reduced to a state of idiotcy, were regarded as having reason enough to conduct themselves with tolerable propriety, and made responsible for their actions to a degree that would startle the criminalists of our own time, ready as most of them are, to look upon the plea of insanity as the last resort of ingenious counsel. Sir M. Hale declared, many years after Lear was written, that insanity affects only the strength and capacity of the mind, and upon this idea he has founded a test of responsibility. 'Such person as labouring under melancholy distempers, hath yet ordinarily as great understanding as ordinarily a child of fourteen years, is such a person as may be guilty of felony or treason.' These views, it is true, belong to a province of insanity somewhat remote from that which engaged Shakespeare's attention, but there can be no difficulty in inferring from his delineations of the disease, in what light he would have regarded them. Can we suppose, for instance, that if the question of the responsibility of Hamlet for killing Polonius had been referred to him, he would have pronounced him guilty of murder in the highest degree, because he possessed more understanding than a child fourteen years old? Had the jurist, in forming his opinions on this subject, meditated upon the pictures of Shakespeare as well as the principles of Coke and Lyttleton, it would have been better for his own reputation, and better—ah! how much better—for the cause of humanity. Would that we were able to say that the Courts of our times have entirely avoided this error, and studied the influence of insanity upon human conduct more by the light of Shakespeare and of Nature, than of metaphysical dogmas and legal maxims." P. 332.

### Dr. Brigham on the Medical Treatment of Insanity at the New York State Lunatic Asylum.

Dr. Brigham having been frequently questioned upon this subject supplies a short paper to the present volume of the "Journal." He observes that, as no specific treatment exists, each case is submitted to remedies appropriate to its stage, *recent cases* usually requiring a mild antiphlogistic treatment. Much depends upon the cause. Thus, if the disease arises from any direct physical injury to the head or violent mental commotion free bleeding and purging are indicated. Such cases, however, are seldom seen in lunatic asylums; and only four out of the 622 patients were bled during the last year, in one of whom only did benefit seem to accrue. Cerebral excitement is occasionally treated by local depletion; but generally by placing the feet in warm water, applying cold to the head and the administration of purgatives. One of the most certain means of subduing maniacal excitement is the pouring cold water from a height of four or five feet directly upon the head; but it must be gently executed, only for a short period, under the immediate superintendence of the physician, and never when the bowels are confined or the stomach full. The warm bath, continued for half an hour or longer, cold being simultaneously very gently applied to the head, exerts also a very calming effect in many cases. In most cases of insanity warm bathing is beneficial; and cold bathing and the shower-bath might be resorted to more frequently, but that the patients are apt to regard them as intended for punishment.

*Emetics* and *cathartics* having proved of little service are now seldom prescribed, guarding against constipation, however, by diet and laxatives. In some recent cases Croton oil has proved very efficacious, apparently effecting a cure. It is the easiest of all medicines in its administration and has led to no unpleasant results.

*Blisters, issues,* and especially *setons* in the neck, have been frequently tried, but rarely effected anything but incidental good by diverting the attention of the patient from imaginary sufferings and delusions.

Great success has frequently attended the use of *opium*. In some cases it appears useless and in a few injurious, especially when the skin is hot and dry, and the pulse full and hard. Although not perhaps frequently curative it is a valuable adjuvant, securing an advantageous calm not otherwise procurable. "In some cases, however, it seems of itself to effect a cure; of this we can have no doubt, after having seen many patients apparently recover while taking it freely, and immediately relapse on its being withheld, and again recover under its use, and finally, after continuing it for a considerable time and gradually diminishing the dose, recover and remain well for years without it." Dr. Pritchard, who formerly spoke very disparagingly of this remedy, in a late edition highly commends it. The following formula is employed in many nervous, sleepless, and hysterical cases. ℞. *Trœ. Lupuli.*; *Trœ. Hyoscy.* āā ℥iv.; *Camphorœ* ʒj.; *Ol. Valerian.* ♏xxxij. Dose 1 to 2 drachms. The following is also useful in some cases of violent mania, especially, as is often the case, when the secretion of urine is defective. ℞. *Trœ. Digital.; Trœ. Scillœ,* āā ʒss.; *Vin. Ant. Tart., Spt. Nitrœ Dulc.* āā ℥j. M. Dose 30 to 60 drops.

Many cases, particularly if they have been of long duration, require *invigorating diet and tonics;* and many patients are brought to the institution seriously injured by the depletion which their maniacal excitement and great muscular efforts have unfortunately given rise to. The usual tonics are prescribed; but from none is a greater advantage derived than the following combination. *Extract. Conii* ℥vj.; *Ferri Carb. Prœcip.* ℥xij.; *Molasses, Wine, Warm Water,* āā *two quarts; Ol. Sassafras* ʒij. *dissolved in Alcohol* ℥viij. M. The usual dose is ½ oz. ad 1 oz. adding a little Tr. Aloes and Myrrh to each dose when a laxative is required. The following combination also has been found "quite serviceable" in many cases of debility and loss of appetite. ℞. *Trœ. Cinchon. Co.* ℥j.; *Trœ. Gentian.* ℥iij.; *Trœ. Capsici* ʒij.; *Sulph. Quin.* ʒss.; *Acid. Sulph.* ♏xv.—M. A dram to be given in water or ginger tea.

Insanity is often complicated with other affections. *Nocturnal emissions* are not infrequent and are very injurious, for which large doses of the *Trœ. Ferri Muriatis* seem the best remedy. So, too, *Passive Menorrhagia,* which is often obstinate and seemingly sometimes a cause of the insanity, may be treated by the same, or better still by *Tr. Cinnamom.* and *Tr. Aloes,* of each from 20 to 30 drops.

"It should ever be borne in mind that disease in the insane is very apt to be masked,—that serious disease of the lungs, or of some of the abdominal viscera may exist, but without being manifested by the usual symptoms, and may therefore be overlooked without careful examination. In other respects not particularized in these remarks, we are not aware that the diseases of the insane require different treatment from those of the sane."

Dr. Brigham's Report of the New York State Lunatic Asylum contains some interesting remarks on some of the physical characteristics of the insane.

*Frequency of Pulse of the Insane.*—Dr. Brigham has recorded the frequency of pulse in 1234 cases, giving the following results. From 40 to 50 in 8; 50 to 60 in 22; 60 to 70 in 183; 70 to 80 in 233; 80 to 90 in 466; 90 to 100 in 144; 100 to 110 in 124, and 110 to 120 in 54. Examining the pulse in 76 sane persons he found it from 60 to 70 in 6; from 70 to 80 in 47; and from 80 to 90 in 23. Both the sane and insane individuals were in a calm state and sitting posture. "Age seems not to have much influence upon the rapidity of the pulse, as a few of the most aged were found to have a rapid pulse. Those who have recently become insane, most generally have a frequent pulse, above 80 in a minute, though there are exceptions to this, as in some such cases the pulse is remarkably slow."

*Size and Shape of the Head.*—Dr. Brigham instituted careful admeasurements of the heads of 1163 insane persons (604 men and 559 women) and of 82 sane persons (45 men and 37 women). They were measured around in three directions: 1, around the head, its greatest circumference; 2, from the opening of one ear, over the head, to the other; 3, from the root of the nose or lower part of the forehead, to the nape of the neck or occipital protuberance. These two last were found to be nearly alike in the same individuals; and, with few exceptions, the heads which were largest in one direction were so likewise in the other. In eight only of the 604 insane men did the largest circumference extend to 24 inches, and the other directions to from $14\frac{1}{2}$ to $15\frac{1}{2}$. In 314 the largest circumference was more than 22 inches, and in 282 from 21 to 22 inches. In 5 of the 45 sane men only did this extend to $23\frac{1}{2}$ inches, and in 18 of the number it was less than 22—so that no material difference was observed in the size of the sane and insane head. In both insane and sane women the highest figure reached by the largest circumference was $22\frac{1}{2}$ inches. In 350 of the former, and 19 of the latter, it was less than 22 inches.

*Weight of the Insane.*—Dr. Brigham likewise presents us with tables of the weights of 1007 insane persons, and believes this point has been too much overlooked. We do not consider any importance is to be attached to any such account of the absolute weight of the insane—every possible latitude of this being perfectly consistent with complete health of body and mind; but we quite concur with him in the following observations upon the indications to be drawn from variations in the weight subsequent to admission.

"We have practised weighing each patient upon admission and occasionally afterwards, and I think we have derived considerable advantage from this custom. It is sometimes a valuable guide in prognosis, and often affords amusement and encouragement to the insane themselves when they find, contrary to their strong convictions, that instead of losing, they are increasing in flesh.

"A majority of the insane, when committed to our care, are less fleshy than natural. They have become more or less emaciated by disease, or by their imaginary troubles. Usually they regain flesh when they begin to recover, and frequently weigh more after complete restoration than at any other period of their lives. Some in the course of a few months have gained from 30 to 40 pounds. In recent cases of insanity we usually predict recovery when patients begin to increase in flesh, especially if at the same time there is some improvement of the mind. On the contrary, where the digestion and sleep and appetite are natural, and the patient increases in flesh, without any diminution of insanity, there is little hope of recovery: also, if the appetite continues good and emaciation increases, there is reason to fear an unfavourable result."

We have often thought that practitioners are too neglectful of the information which statical data would furnish to them. Every one must have at times felt the great desirableness of being assured whether the patient is gaining or losing flesh; while the indefinite and frequently inaccurate statements furnished upon this head (especially by women who are continually confounding mere distension with increase of substance) may often mislead. In all chronic cases, a correct register of the patients' weight, as ascertained at periodical intervals, should be kept, the timid or wavering not being necessarily made acquainted with the results.

*Hereditary Predisposition.*—This, Dr. Brigham believes, exerts more influence in the production of the disease than all other causes combined.

"It does not of itself excite the disease, but when it strongly exists, a trivial cause will develop it. Thus, most of the supposed exciting causes in the foregoing table would, of themselves, be inoperative, if there was not an inherited constitutional tendency to insanity." The children may escape, and the grand-children suffer. Careful enquiry has shewn that insanity is a little more likely to be transmitted by the mother than the father, and that the mothers are considerably more likely to transmit it to daughters than to sons; while the fathers most frequently transmit it to sons. Thus of 79 men, 42 had insane fathers and 35 insane mothers, and in two both parents were deranged; and of 96 women, 37 had insane fathers and 56 insane mothers, three inheriting the predisposition from both parents. "When children resemble in personal appearance the insane parent, and manifest the same peculiarities of feeling and temper, there is reason to apprehend they will be more or less disposed to the disorder of the parent they resemble." Of 1181 patients (594 m., 587 w.), 315 were known to have insane relatives, and in 175 cases the parents were the relatives. Many others would probably evidence the same predisposition were their history more accurately known. Dr. Brigham has, however, contrary to the opinion of most, found the inherited form of insanity as curable as any other, but very liable to relapse from slight and various causes. Individuals so predisposed may have repeated attacks, each one from a different exciting cause. The education of individuals having this predisposition is of vast importance.

"The early education of all such requires much attention. Great pains should be taken to form a character not so subject to strong emotions, to passion and caprice. Among the most frequent causes of insanity in those not predisposed to it, is the over-indulgence of the appetites and passions in early life; and to those who inherit a tendency to this disease, such a course is highly pernicious. The utmost attention should be given to the securing a good bodily constitution. Such children should be confined but little at school; they should be encouraged to run about the fields and take much exercise in the open air, and thus ensure the equal and proper development of all the organs of the body. They should not have the intellect unduly tasked. Very early cultivation of the mind, and the excitement of the feelings by the strife for the praise and the honour awarded to great efforts of mind and memory, are injurious to all children, and to those who inherit a tendency to nervous diseases or insanity, most pernicious."

*Suicidal Form of Insanity.*—Of 1181 patients admitted into the New York Asylum, 156 (63 m. and 93 w.) were disposed to suicide. "It is however a consoling fact, that this alarming variety of insanity is quite often a curable one. Among the most complete and permanent recoveries we have ever known, are a considerable number who, for several months, were very strongly inclined to self-destruction."

### *Homicidal Insanity.*

The Report contains many interesting observations upon this all-important subject. Dr. Brigham believes the homicidal insane may be arranged into six classes, differing in their mental condition, as indicated by the motives which actuate them, or the circumstances which accompany the deed. So unsettled are the views of the public and of the legal and medical professions upon this subject, that we are glad of the opportunity of recording any remarks of so acute and experienced an observer; and certainly the first step towards a more accurate comprehension of these cases would seem to be a more distinct apprehension of their distinguishing traits. The classes indicated by Dr. B. are as follow. 1. *Those who take life in a paroxysm of insane passion or fury.* Insanity so frequently induces excessive irritability of temper, that every asylum contains patients who are disposed to assault or kill. Most of the cases related in the newspapers of insane persons at large killing their relatives or friends during a gust of passion belong to this class. In general, other acts have previously indicated insanity on the part of the perpetrator; although occasionally cases occur in which it is difficult to decide whether the act results from insanity or depravity and violent passion.

"Usually, however, in these somewhat doubtful cases, it will be found that those who ought not to be considered responsible for their actions, by reason of insanity, have suffered from some severe illness, or from some great mental disturbance, since which time their temper and disposition have undergone a marked change. When no such facts are found to exist, nor any other evidence of mental disorder, we think the act itself committed during violent passion, is not proof of insanity, though it may have been committed under such aggravated provocation as to render it, in public opinion, justifiable homicide."

In the *second* class are placed those *who commit a homicide from delusion,* who are deceived or misled by their hallucinations, illusions, or disordered imaginations; and this is the form of homicidal insanity which is more prevalent than any other; the actuating motive being often benevolence towards the victim sacrificed to the delusion.

The *third* class is made up of "those who kill indiscriminately and apparently from a love of taking life; from a diseased propensity and conscious desire to destroy others, against which act neither reason or conscience remonstrates." Some of those persons seem almost constantly desirous of injuring or killing others: they are conscious of this; but are actuated by no motive, malice, or passion.

In the *fourth* class we have persons "who kill without any apparent motive, from a sudden impulse, but of which they are not conscious, and who retain no recollection of anything that prompted them to the act. Those belonging to the *third* class, have an intense desire to kill, of which they are conscious, and are usually evidently insane in other respects; but those belonging to the *fourth* class kill from a sudden impulse, without any desire or conscious feeling relating to the act, and not unfrequently *the act itself is the first noticeable evidence of their mental derangement.*"

The *fifth* class is composed of persons who commit the crime without motive, from an irresistible impulse, of which they are conscious, and against which reason often remonstrates. Gall, in his work upon the brain, instanced several cases in which the insane implored that they might be protected against the effects of their propensities, and similar ones abound in works upon insanity.

To the *sixth class* belongs such as kill from imitation, or an insane love of notoriety.

After this analysis of the cases that have come under his notice, Dr. Brigham adverts to the difficult question of legal responsibility, concerning which, opinions seem to be as unfixed and practice as uncertain in the United States as in our own country. These cases, in fact, often form the most embarrassing ones which can come before a jury, even now that the public and the legal profession have acknowledged, in many instances, the existence of moral insanity; for, while humanity forbids the condemnation of an unfortunate individual whose only crime is disease, the protection of the public from the consequences of a too easy reception of proofs of the existence of this, is a duty no less urgent, and humanity no less genuine. The present strong feeling prevailing in this country against the validity of the punishment of death even in cases of murder, contributes another element of difficulty—juries finding insanity in many cases in which they otherwise would not, were they not fearful of entailing death as a consequence. So much is public opinion divided upon these questions, that it is seldom that the decisions of our tribunals give that general satisfaction, which is so commonly the case in relation to other subjects.

"The great difficulties and mistakes that have occurred on this subject," Dr. Brigham observes, "have arisen, we believe, from courts attempting to define, what cannot be correctly defined, viz. insanity; and to establish what does not exist, a certain test of insanity by which the existence of this disease can be recognized. This statement, we think, is fully confirmed by a brief history of the attempts of courts to define insanity, and to furnish juries a rule to guide them in determining the question of responsibility."

The old doctrine, as laid down by Coke and Hale, was, that for exemption on the ground of insanity, a *total deprivation of memory and understanding* must exist. Erskine admitted this on the trial of Hatfield in 1800; but his client was not in this predicament, and was acquitted on the ground of the existence of *delusion,* this being therefore established as the true character of insanity. However, it did not save Bellingham ten years later, who, had he been tried in our own times, would have certainly been acquitted as insane. The test laid down on that occasion by Gibbs, and admitted by Mansfield, was the ability of the accused to distinguish right from wrong, even though he might be incapable of conducting his own affairs.

"Since then the law, that is the decisions of the higher courts of England, have been loose and fluctuating. Generally the ability to distinguish right from wrong has been insisted upon more or less strongly. Not unfrequently, however, it has been partially or entirely abandoned, as it must have been on the trial of M'Naughten for killing Mr. Drummond in 1843. In this case it was found that the prisoner had good memory and understanding, and was capable of transacting business correctly, and there was no evidence that he did not understand the distinction between right and wrong at the time he committed the act for which he was tried and justly acquitted.

"Within a few years the English House of Lords have endeavoured to obtain from the Judges of the Law Courts their views of the law relating to insanity, or rather a legal definition of insanity, which should henceforth furnish a rule for the guidance of courts and juries whenever in criminal cases the plea of insanity might be set up. The answer of the Judges to the questions propounded to them must be regarded as a signal failure in this respect. Their answer was in fact but the repetition of the dictum we have mentioned, *that a man is responsible if he is capable of knowing right from wrong.* As was predicted at the time it was disregarded immediately after, as it had been in M'Naughten's case just before. It was not at the time satisfactory to some of the most enlightened members of the House of Lords. Lord Brougham complained that the test was vague and unsatisfactory, and doubted whether the juries before whom the question is tried really comprehended what is meant by it. It has been disregarded in England in numerous instances since."

Certainly the solemn assemblage of the judges which took place on the occasion in question was one of the happiest examples of the Mountain in Labour which modern times have afforded. That conclusions in which nothing was concluded should follow the deliberations of a body of men so little accustomed to an enlarged contemplation of human nature, and so religiously respectful of precedents and technicalities, is far less surprising than would have been the laying down any intelligible and comprehensive principle.

The decisions in the U. S. Courts alluded to by Dr. Brigham, seem to be nearly as uncertain and unstable, as guides for the future, as our own. In reference to the test already alluded to, he adds :—

" Although judges appear to think that it is easy for juries to decide whether the prisoner was able to distinguish right from wrong at the time, we are of opinion that there are few more difficult. Separate from the fact that no being short of Omnipotence always knows right from wrong, and that what is right in one age or nation is considered wrong in another, *how* are the jury to decide that, at the precise moment of committing a homicide, a man can or cannot distinguish right from wrong? What kind of evidence establishes this? The legal presumption is, that a man does know; but in case he does not, how is this to be proved? We certainly do not know, and believe, with Lord Brougham, that 'juries do not really comprehend what is meant by the question.' * * *

" We have endeavoured at various times to ascertain from the insane themselves their ability to distinguish right from wrong, and for this purpose have questioned on the subject in various ways several hundreds, and we cannot better exhibit its inapplicability as a test of their responsibility, than to say what the truth enables us to say, that a large proportion of the insane now in asylum appears to understand its distinctions as well as persons in the community at large. We also believe this to be true of most of the insane we have seen elsewhere; and we confidently assert that this will be found to be the opinion of every person who has had the charge of an institution for the insane, and seen many deranged persons. But the cases we have already mentioned show the utter worthlessness of this as a test of the responsibility of the insane. One of the women killed her own child for the purpose of procuring her own death by execution. She certainly knew she was doing a wrong act, and one for which she expected to be punished. Others commit homicide from a sudden impulse, who have not previously exhibited any wrong conduct or intellectual aberration. A mother kills her only child, or a man his wife and family, to whom he is known to be ardently attached; or a child a parent, as in the instances we have mentioned. To such cases none of these tests of insanity apply, and yet they are among the most palpable and unquestionable cases of that kind of mental disease which should be an excuse for criminal acts.

" Notwithstanding the great improvement within the last fifty years, that has been made in the treatment of the insane, owing to an increased knowledge relating to insanity, it is still a fact that some of the most deplorable forms of this disease are not even now allowed to annul responsibility. Although it is the law of this and every civilized country, that it is the reason of man which makes him accountable for his actions; and that the deprivation of reason acquits him of crime; yet it is a lamentable fact that, at the present time, courts of justice do not acknowledge the existence of some of the worst forms of mental alienation, and which, in the opinion of those acquainted with the insane, are as well established as any other. In the words of an enlightened Judge of this State, 'the law upon the subject of Insanity in its slow and cautious progress still lags far behind the advance of true knowledge.' * * * * * * * * Far from us is any desire to skreen the guilty by the plea of insanity, and no one can regret more than us to see this plea improperly set up, as this must tend to jeopardize it when justly made. But we cannot resist the conviction that, by the different constructions given by courts to the law of insanity, that the rights both of the insane and of the community may be in danger. That in one case, when popular feeling is much excited against the accused, the strictest rules of law and the severest tests of insanity known to the common law of England are enforced, and notwithstanding strong proof of insanity conviction follows; while in another case, with little proof of insanity, but with popular sentiment in favor of the prisoner, other constructions are given to the law, and he is acquitted."

We have quoted much more largely from this Report than is customary with us in respect to similar documents: but the contents, as our readers may now judge, are of no ordinary value.

### M. Brierre de Boismont on Establishments for the Insane in Belgium, Holland and England.

The opinions of foreigners upon our institutions when matured by sufficient information and delivered with candour, are always valuable and acceptable, confirmatory as they frequently are of advantages already enjoyed or suggestive of desirable improvements. M. Brierre, already well known to our readers as an able practitioner in this department of medicine, has recently paid us a flying visit, and has published in the "Annales d'Hygiene," the impressions which his inspection of the metropolitan asylums produced. Before noticing these, however, we may advert to the account he furnishes of the singular Belgian establishment at *Gheel*, which he visited during the same tour. He reports that the provisions for the insane in Belgium and Holland are lamentably defective and far behind those of the rest of Europe in all the appurtenances for comfort and treatment.

*The Insane Colony at Gheel.*—Gheel, although still termed a village, is well entitled to the appellation of a town, inasmuch as it numbers from 7 to 8000 inhabitants, and is now a flourishing place, forming a marked contrast with its wretched condition as described by Esquirol a quarter of a century since. The entire number of lunatics dispersed over the village and adjoining hamlets amounts to about 800; the sum paid for them varying from 624 to as low as 24 francs per annum. Taking the mean of the entire number, the daily cost per head is only half-a-franc. The sum fixed by authority is from 170 to 200 francs per annum. A very limited number who live with the better classes of citizens pay from 15 to 1800. On examining the abodes of the peasants in which the lunatics resided, M. Brierre found them clean and comfortable, one patient only for the most part being consigned to each house. As the peasants are very anxious to retain these guests, and consider their removal at the instance of the Inspector casts a stigma upon them, it becomes their interest to treat and feed them well. There is, however, no special place for the treatment of the patients, and no medical treatment, save for incidental maladies, is put into force, while coercion is employed in certain cases to an extent utterly discountenanced in the rest of Europe. Thus, although the mass of the patients have liberty to go out and in when they choose, and to walk as freely about as the sane, M. Brierre observed some of these had heavy rings on their legs or hands just like galley-slaves, such having heretofore made attempts at escape : so too in the houses he observed large iron rings for the attachment of restive patients. At the time he visited Gheel there were not more than twenty under any restraint, and this was above the usual number. He heard no cries of suffering, even during the silence of a very hot night. Several of the patients pursued occupations, the majority however being unwilling or unable to work. The sojourn of these patients does not seem to have operated injuriously upon the inhabitants of the place, who become very rarely insane, and that usually from the ordinary causes. The promiscuous intercourse of the insane of opposite sexes also has not given rise to the production of illegitimate births—a circumstance which the author explains by the active surveillance the peasants maintain, and the moral and phlegmatic character of the Flemings. It is evident that, although the lunatics are well and kindly treated, just as if they were members of the respective families they reside in, there their advantages end. No attempt at physical or moral treatment is made, and no appliances for these exist; and the colony can be looked upon as little else than a comfortable abode for incurable patients.

" However we may reply to the questions of what are the advantages or inconveniences of this establishment; whatever benefits accrue from it, and whatever may be its future destination, we at least cannot but remark that, while the insane in France & England and many other countries, were imprisoned, chained and buried in cages and huts just like wild beasts, there had existed for ages in a lonely and unknown part of Belgium an establishment where hundreds of the insane were free and abandoned to themselves. Simple good-sense had revealed to these poor peasants that mildness and kind treatment should form the basis of their relations with this strange description of guests, and that force should not be resorted to save in case of danger."

*The English Asylums.*—Dr. Brierre's attention was confined to Bethlem, St. Lukes's, Hanwell, and one or two private asylums. His general opinion of *Bethlem* is highly favorable, save in respect to its situation within the metropolis. Among the few observations he makes of a qualificatory character are the following.

"There is nothing particular in the medical treatment adopted. In the galleries there is a small apartment with the warm, cold, and surprise baths; but its narrow dimensions would only admit of one or two being bathed at a time; and we have the full conviction that this means, although employed, is not so frequently applied as in France. Medicinal substances seem to constitute a large portion of the treatment, and I do not wish to deny their importance; but attendance upon great numbers of the insane leaves no doubt in my mind of the greater importance of baths in this affection; and I have elsewhere* shown that, by their aid, we can abridge the duration of certain forms of insanity, but for this end many baths and appliances are requisite.

" At the present day, when the rules which should govern this description of establishments are agreed upon, there is no physician who would not admit that, ere long, this of Bethlem must be removed away from the metropolis to some agreeable locality. Another important amelioration would be the *residence of the physicians in the establishment itself;* but for this purpose a liberal salary should be accorded to them. Another measure, no less desirable, would be the appointment of certain special pupils, who should pass several years within its walls, and for whom it would be but just to secure appointments to provincial asylums upon their leaving this one. The conduct of France in this particular seems to me deserving of imitation; for almost all the asylums founded by the law of 1838, have been entrusted to physicians, whom M. Ferrus, Inspector-General of Asylums, has selected from those who have exclusively devoted themselves to the study of mental disease.

" The number of patients to be consigned to the care of the physician is not a matter of indifference: and if we consulted our own experience we would limit this to 100, 70 chronic and 30 acute cases."

Dr. Brierre gives a somewhat detailed account of the Criminal Wing at Bethlem, as being likely to prove most interesting to his readers in France, where it seems no such provision for the separation of this description of lunatics prevails. At the period of his visit there were 97 patients (77 m. and 20 w.), the causes of whose detention are thus distributed. Crimes against the State, 2. Crimes against the person, 63. Crimes against property, 32. " I may be mistaken, but I have the conviction that the prospect of a sojourn in such a place would be more likely to deter a true criminal than the perspective of the scaffold."

Quite agreeing with the author, that the Criminal Lunatics should be separated from those of other descriptions, we would carry the principle farther still, and have a separate establishment under the control of Government, erected for them in a part of the country remote from the metropolis. Their detention at Bethlem is mischievous to the other patients, by reason of their more noisy dispositions, the prison-like aspect they give the place, and the limitation of accommodation they entail. It is no less injurious to the public interests. Located in an establishment under the governance of the city authorities who have free ingress to it, they become a sort of raree show to all the fashionable idlers and visitors of the metropolis, for whose morbid appetites our worthy citizens have ever shown themselves most desirous of catering, whether by the exhibition of the poor wretches alluded to, or by the revolting proceedings at the condemned sermon or condemned cell in Newgate. It is true public opinion has of late sternly condemned these procedures, but we have no security of their non-revival but the removal of such high trusts from such incompetent hands. The mischief done to the moral sense of the community, and the stimulus given to the morbid desire of imitation so rife in the

---

* For an account of M. Brierre's opinions of the curative agency of prolonged warm baths in acute insanity, see Rev. Medicale, Nov. 1847, and Med.-Chir. Rev. N. S., Vol. V., p. 282.

insane and in those verging on insanity, by the recital in the newspapers of the various circumstances alluded to, should long since have secured the interference of the State.

While praising the great cleanliness (indeed he passes great encomia on this important item of management, as manifested by the utter absence of bad smells, in all our establishments) and the general good management at *St. Luke's*, M. Brierre properly maintains that, in so limited a space and so confined a situation, it is impossible for the patients to profit by the progress of improvements in this branch of medical science to the extent they otherwise might do. He also much objects to patients being allowed to sleep in separate rooms, being persuaded that efficiency of supervision and inspection is best secured by dormitories containing from eleven to twelve beds.

After describing Dr. Philp's private asylum at Kensington, he observes—

"I have examined Dr. Philp's house with much attention, and in regard to arrangement, cleanliness, and comfort, it calls for, in my opinion, nothing but praise; but I must avow that my sympathies are enlisted with that system in which we live continuously with our patients. It is my conviction that the physician and his family should reside in the midst of the insane, so as to have them incessantly under the eye, and receive into his intimate society the convalescent, the monomaniacal, and especially the melancholic. This is my system, in which I am so ably seconded by the devotion of my wife, who keeps those poor suffering souls in her apartments for ten hours together, and it appears to me the means which, united with reading, music, diversions, exercise, and employment, furnishes the most incontestable results."

*Hanwell.*—This establishment M. Brierre visited with much curiosity and interest, as the great scene of the development of the non-restraint system. To this he has always shown himself an enlightened opponent; believing, in common with many other observers in this and other countries, that however infrequently it may be required, there are cases in which its mild application becomes the greater kindness to the unfortunate lunatic. There can be no doubt that the experience of Hanwell has been attempted to be prematurely generalized in respect to establishments receiving a different description of patients.

"The aspect of the inmates of Hanwell, the information I have obtained in England, and that which was furnished me in the establishment itself, have left me in no kind of doubt as to the description of patients which this asylum is filled with. It is not to be dissimulated that Hanwell is a true hospital of incurables. Almost all the patients brought to it have already been treated elsewhere, and are consequently in a very favourable disposition for the non-restraint system. To convince ourselves of this, it suffices to observe what takes place in our own establishments. At what epoch do paroxysms of fury, cries, agitation, suicidal ideas, the desire to injure others, refusal to eat, or attempts at escape, take place? At the commencement of the disease. It is then only that we are obliged to place the insane in the baths by force, to apply the strait-waistcoat, to fix them in their chairs, or to imprison them in their cells. But this condition lasts a very short time: and never does the excitement of acute mania resist seven or eight prolonged baths. Soon all this effervescence and fury subside; and patients, who seem determined to brave all measures, become tranquil, and follow the customs of the establishment. When new paroxysms occur, they are, most commonly, but transitory: a bath, or a seclusion for a few hours, or the privation of some favourite dish, sufficing to restore order.

"The conditions here are quite different to those observed in insanity at its commencement. Moreover, we should take into consideration the characters of the English people. In England people are brought up with a respect to the laws, a deference for the upper classes, and a submission to custom. There they are slaves to the letter, and individuality, controlled by religion and patriotism, is not incessantly opposing itself to the slightest obstacle. Do not these national differences, joined to the chronic or incurable condition of the patient, suffice to a certain extent to explain the success of the non-restrain system at Hanwell?

"It has been said, and not without foundation, that solitary confinement in a padded cell, and the subjection of a patient by a greater or less number of attendants are truly coercive measures, the name of which has alone been changed. But even such means are not always applicable. 'For those who know the insane,' says M. Falret, 'non-restraint is a fiction, a simple substitution of one means for another;' and I may add my profound conviction, that solitary confinement is a mode of repression a thousand times more painful and more restrictive of liberty than the strait-waistcoat; and that it is contrary to the first principles which should prevail in the treatment of the violent insane, which consist in placing them in conditions the most favourable for taking that exercise in the open air, which Nature so imperiously demands for them."

M. Brierre de Boismont thus concludes his papers:—

"In summing up my impressions it will be seen—1. That the English establishments I visited are remarkable for their size, cleanliness, and the care bestowed upon the patients; but that they differ from those of France in their construction, classification, and mode of treatment. In our comparative examination we should not lose sight of the serious, methodical, puritanical, matter of fact, but comfort-loving character of the English nation. 2. Some of these are defective in space, and consequently in the means for those agricultural labours, which form an indispensable portion of all effectual treatment. Their position in the midst of towns is also an obstacle to their amelioration. 3. The separation of the Criminal Insane is useful, and I sincerely desire to see it adopted in France with certain modifications. 4. The crowding together of patients, so as to assemble 1000 in one establishment, as at Hanwell, is contrary to the objects of Asylums. 5. The increase of incurable patients which threatens the invasion of the best institutions, should induce measures for the separation of such from those who have yet a chance of cure. 6. The non-restraint system has rendered good service by forcibly directing attention to barbarous systems of treatment in complete disaccordance with the manners of our times, but it is not always applicable."

